# Cortical connectivity maps reveal anatomically distinct areas in the parietal cortex of the rat

**DOI:** 10.3389/fncir.2014.00146

**Published:** 2015-01-05

**Authors:** Aaron A. Wilber, Benjamin J. Clark, Alexis J. Demecha, Lilia Mesina, Jessica M. Vos, Bruce L. McNaughton

**Affiliations:** ^1^Canadian Centre for Behavioural Neuroscience, The University of LethbridgeLethbridge, AB, Canada; ^2^Department of Neurobiology and Behavior, University of CaliforniaIrvine, CA, USA; ^3^Department of Psychology, The University of New MexicoAlbuquerque, NM, USA

**Keywords:** posterior parietal cortex, retrosplenial cortex, connectivity analysis, automated tracing, cortical flat maps, connectome, segmentation, thalamus

## Abstract

A central feature of theories of spatial navigation involves the representation of spatial relationships between objects in complex environments. The parietal cortex has long been linked to the processing of spatial visual information and recent evidence from single unit recording in rodents suggests a role for this region in encoding egocentric and world-centered frames. The rat parietal cortex can be subdivided into four distinct rostral-caudal and medial-lateral regions, which includes a zone previously characterized as secondary visual cortex. At present, very little is known regarding the relative connectivity of these parietal subdivisions. Thus, we set out to map the connectivity of the entire anterior-posterior and medial-lateral span of this region. To do this we used anterograde and retrograde tracers in conjunction with open source neuronal segmentation and tracer detection tools to generate whole brain connectivity maps of parietal inputs and outputs. Our present results show that inputs to the parietal cortex varied significantly along the medial-lateral, but not the rostral-caudal axis. Specifically, retrosplenial connectivity is greater medially, but connectivity with visual cortex, though generally sparse, is more significant laterally. Finally, based on connection density, the connectivity between parietal cortex and hippocampus is indirect and likely achieved largely via dysgranular retrosplenial cortex. Thus, similar to primates, the parietal cortex of rats exhibits a difference in connectivity along the medial-lateral axis, which may represent functionally distinct areas.

## Introduction

The ability to find our way through complex environments and interact with them is generally thought to involve the use of multiple stimulus sources and frames of reference (O'Keefe and Nadel, 1978; Gallistel, 1990; McNaughton et al., 1991). For instance, movements that are based on environmental cues have been characterized as involving an initial egocentric mapping of the perceived location and orientation of objects relative to oneself, and a subsequent remapping to a world-centered (i.e., allocentric) framework, allowing a subject to act upon objects, or move to particular goals in relation to them. This allocentric frame of reference is a central feature of “cognitive mapping” theories (O'Keefe and Nadel, 1978) based in large part on the finding that hippocampal neurons form a population code of spatial location in an environment (McNaughton et al., 2006; Moser et al., 2008). Recent evidence indicates that, in addition to encoding particular places, neurons in the hippocampus (O'Keefe and Burgess, 1996; Deshmukh and Knierim, 2013; Wilber et al., 2014) and parahippocampal cortex (Hartley et al., 2000; Lever et al., 2009) fire at specific distances and directions relative to salient environmental cues, suggesting that the hippocampal formation may be involved in the storage of “landmark vectors” defining the direction and distance between environmental cues and goals (McNaughton et al., 1995; Byrne and Becker, 2007). The parietal cortex (PC), which rests between visual and sensorimotor systems, may be involved in the first stages of vector-based computations in relation to landmarks. This follows the long held view that the PC contributes to the encoding of an egocentric coordinate system based on some portion of the body such as the retina, head, or somatosensory system (McNaughton et al., 1994; Xing and Andersen, 2000; Wolbers et al., 2008; Howard et al., 2013; Schindler and Bartels, 2013; Spiers and Barry, 2015). Supporting this hypothesis, a recent study in our laboratory demonstrated that caudal regions of PC, commonly referred to as secondary visual cortex (i.e., V2MM and V2ML), contain neurons that are modulated by the egocentric direction of a landmark, allocentric head direction, and the conjunction of both firing characteristics (Wilber et al., 2014). Other studies have identified a role for the rodent PC region in processing route-centric information such as the progress made along a path in a complex maze (Nitz, 2006, 2012), and the anticipation of movements (Whitlock et al., 2012), a prominent feature in cells that have conjunctive responses for egocentric and allocentric information (Wilber et al., 2014).

The conclusion that the PC serves a broad role in coordinating egocentric and allocentric relationships with environmental objects implies that this region has a broad input-output relationship with motor, sensory, and limbic brain regions. However, rodent electrophysiological and behavioral studies targeting or manipulating PC function have typically been conducted with almost no consideration of possible anatomical variation in connectivity and cytoarchitecture. In addition, recent attempts to map the cortical connectome in mice (Wang et al., 2012; Oh et al., 2014; Zingg et al., 2014) have provided little information to address possible subregional differences in the cortical-cortical connectivity of the PC region. Specifically, differences or similarities between the anterior regions of the rat PC, commonly referred to as parietal association area (PtA), and the posterior zones termed V2M are not quantitatively studied in these experiments. Similarly, the organization of inputs/outputs along the medial-lateral regions of the PC is not described in either rat or mouse studies. In the rat, the PC can be subdivided into four zones defined by cytoarchitectural differences: a rostral region composed of medial and lateral components (MPta and LPta, respectively; Paxinos and Watson, 2007), and a caudal PC also composed of medial and lateral components (V2MM and V2ML, respectively; Paxinos and Watson, 2007). Thus, in the present study, we set out to map the cortical-cortical and thalamo-cortical connectivity of the entire rostral-caudal and medial-lateral span of this region. To do this, we used custom and open-source image processing software (Bjornsson et al., 2008; Schindelin et al., 2012; Rey-Villamizar et al., 2014) that allowed the automatic detection of neurons filled with retrograde tracer and estimate anterograde projection strength. Neurons that were identified as retrograde tracer filled cells were mapped onto unrolled 2D cortical maps for entire brains, and the densities for cortical and thalamic regions of interest were evaluated across the anterior-posterior and medial-lateral span of the parietal cortex.

## Materials and methods

### Subjects

All experiments were performed on 3–15 month male and female Fisher Brown Norway rats (*n* = 18, 170–430 g) and were carried out in accordance with the University of Lethbridge Animal Welfare Committee and conformed to NIH Guidelines on the Care and Use of Laboratory Animals.

### Neuroanatomical tracers and microinjection surgery

Each animal received a unilateral microinjection of either a retrograde or an anterograde tracer into the PC. The fluorescent retrograde tracers, cholera toxin-B Alexa Fluor conjugate 594 (CTB; 1% in 1 × PBS; Life Technologies, Burlington, ON) and Fluoro-Gold (FG; 4% in 1 × PBS; Fluorochrome, Denver, CO), were used to map inputs to the PC. The anterograde tracer, biotinylated dextran amine (BDA; 10,000 MW, 5% in 1 × PBS; Life Technologies, Burlington, ON), was used to map the outputs of PC. Animals were anesthetized with isoflurane, then a small unilateral craniotomy (counterbalanced between hemispheres) was made above the intended injection site (see Table 1 for stereotaxic coordinates). The injection location varied across rats to provide rostral-caudal coverage of the areas traditionally characterized as PC (MPta and LPta, respectively; Paxinos and Watson, 2007) and the area previously characterized as medial secondary visual cortex (V2MM and V2ML, respectively; Paxinos and Watson, 2007), but recently shown to be functionally similar to primate PC (Wilber et al., 2014). Following craniotomy, tracer was loaded into the glass micropipette of a microinjection unit (Nanoject, Drummond Scientific Company, Broomall, PA), and the pipette was centered above the craniotomy. For each rat, the pipette was lowered into the brain and rested for 1 min to allow the tissue to settle. After this 1 min delay three injections of 0.05 uL/30 s were administered. After a total of 0.15 uL of tracer was injected, the pipette was left in place for an additional 1–5 min to allow for tracer diffusion similar to what has been done in previous studies (Kowall et al., 1991; Le Bé et al., 2007; Peters et al., 2008; Wachter et al., 2010). After the injection was complete, the pipette was slowly removed from the brain, the craniotomy was filled with gelfoam, and the skin was sutured.

**Table 1:** **Stereotaxic coordinates of injections targeting PPC**.

**Intended Target**	**Anterior-posterior (mm)**	**Medial-lateral (mm)**	**Dorsal-ventral (mm)**
MPtA/LPtA^c^	−4.0^a^	±2.0 or 3.0^a^	−0.5^b^
V2MM/V2ML^d^	−4.5	±2.0 or 3.0	−0.5
V2MM/V2ML^e^	−5.0 or 5.5	±2.0	−0.5

a *Relative to bregma (A/P: posterior; M/L: lateral)*.

b *Relative to cortical surface*.

c *Injections at these co-ordinates were performed in 5 of the rats for retrograde tracer*.

d *Injections at these co-ordinates were performed in 5 of the rats for retrograde tracer*.

e *Injections at these co-ordinates were performed in 2 of the rats for retrograde tracer*.

### Tissue processing and immunohistochemistry

After a 14-day survival period, rats were overdosed with euthansol and perfused transcardially with a phosphate buffer solution (pH 7.4) followed by 4% paraformaldahyde (PFA) in Phosphate Buffered Saline 0.1 M (PBS). Brains were extracted and post fixed in PFA 4°C for 24 h, then cryoprotected in 30% sucrose (with or without 0.02% sodium Azide). Brains were then embedded in agarose (Agarose-1B, Sigma-Aldrich Canada Co., Oakville, ON) and sectioned in the coronal (*n* = 13) or sagittal (*n* = 5) plane at 50 um using a custom vibratome (Model VT1200 S, Leica Biosystems, Concord, ON; with modifications by Peira Scientific Instruments, Belgium). The vibratome was additionally equipped with a camera mounted above the specimen thereby allowing the acquisition of block-face images for the purpose of registering processed tissue sections and 3D rendering (data not shown).

**Figure 1:**
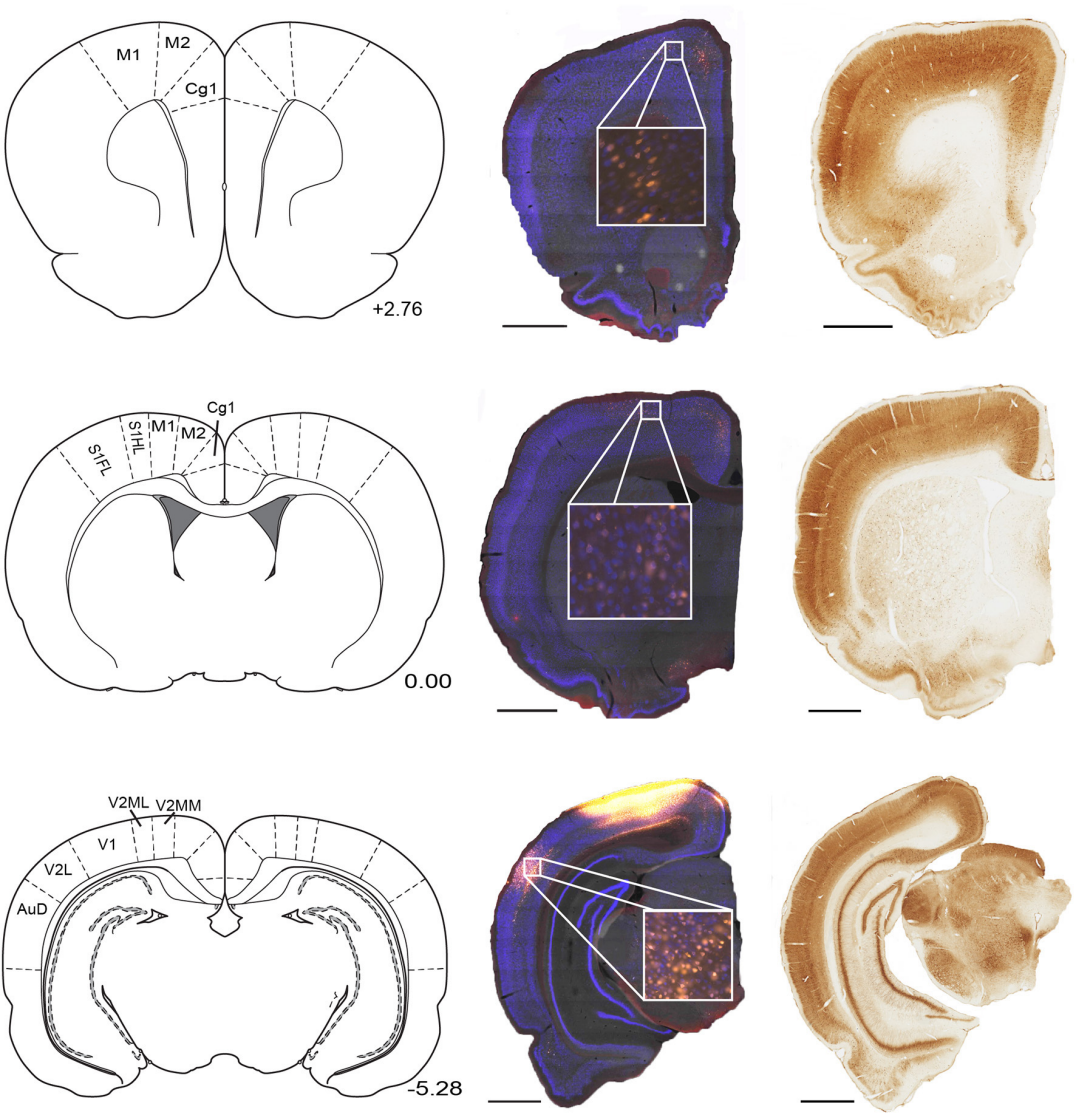
**Parietal cortex receives inputs from regions along the full rostral to caudal extend of cortex**. Example NeuN (middle) and corresponding parvalbumin stained sections (right) taken from three rostral-caudal levels of a brain that received microinjection of fluoro-gold (yellow filled cells) into the parietal cortex. Regions of interest which contained retrogradely-labeled cells are labeled on corresponding panels from Paxinos and Watson (2007). Position with respect to bregma (mm) is indicated next to each panel. Scale bars = 1.5 mm. Abbreviations for Figures 1–10: Anterodorsal thalamic nucleus (AD), Anteromedial thalamic nucleus (AM), Anterior pretectal nucleus (APT), Secondary auditory cortex, dorsal area (AuD), Auditory cortex, all areas (AUD), Anteroventral thalamic nucleus (AV), Cingulate cortex, area 1 (Cg1), Cingulate cortex, area 2 (Cg2), Claustrum (Cl), Central medial thalamic nucleus (CM), Dorsal lateral geniculate nucleus (DLG), Ectorhinal cortex (Ect), Infralimbic cortex (IL), Intermediodorsal thalamic nucleus (IMD), Laterodorsal thalamic nucleus (LD), lateral entorhinal cortex (LEC), Lateral posterior thalamic nucleus, laterorostral part (LPLR), Lateral posterior thalamic nucleus, mediorostral part (LPMR), Lateral parietal association cortex (LPtA), Primary motor cortex (M1), Secondary motor cortex (M2), Mediodorsal thalamic nucleus (MD), Medial Entorhinal Cortex (MEC), Medial orbital cortex (MO), Medial parietal association cortex (MPtA), Parasubiculum (PaS), Paracentral thalamic nucleus (PC), Parafascicular thalamic nucleus (PF), Posterior limitans thalamic nucleus, Prelimbic Cortex (PL), Posterior thalamic nuclear group (Po), Perirhinal cortex (PRh), Paratenial thalamic nucleus (PT), Paraventricular thalamic nucleus (PV), Reuniens thalamic nucleus (Re), Rhomboid thalamic nucleus (Rh), Retrosplenial cortex dysgranular (RSD), Retrosplenial granular cortex (RSG), All primary somatosensory cortex regions (S1), Primary somatosensory cortex, forelimb region (S1FL), Primary somatosensory cortex, hindlimb region (S1HL), Secondary somatosensory cortex (S2), Submedius thalamic nucleus (Sub), Temporal association cortex (TE), Primary visual cortex (V1), Secondary visual cortex, lateral area (V2L), Secondary visual cortex, all medial areas (V2M), Secondary visual cortex, mediolateral area (V2ML), Secondary visual cortex, mediomedial area (V2MM), Ventral anterior thalamic nucleus (VA), Ventrolateral thalamic nucleus (VL), Ventromedial thalamic nucleus (VM), Ventral orbital cortex (VO), Ventral posterolateral thalamic nucleus (VPL).

Sections were collected in 3 parallel series in PBS. For CTB and FG injected brains, one series was processed with a NeuN antibody (a selective neuronal marker; Figure 1) conjugated to fluorescent dye (Alexa Fluor 405; Life Technologies, Burlington, ON), mounted in PBS, and cover slipped with Vectashield (Vector Laboratories, Burlington, ON). A second series was mounted and stained with Cresyl violet. The final series was stained for parvalbumin (Boccara et al., 2010). This procedure involved processing tissue with anti-parvalbumin antibody, incubating the tissue in avidin and biotinylated horseradish peroxidase (Vectastain ABC, Vector Laboratories, Burlington, ON) and lastly subjecting the sections to a diaminobenzidine (DAB) reaction. Tissue was mounted in PBS, dehydrated, cleared, and coverslipped.

For BDA injected brains, one series of sections was incubated in avidin and biotinylated HRP (Vectastain ABC, Vector Laboratories, Burlington, ON) and subsequently stained using DAB with NiCl_2_ intensification. A second series was stained for parvalbumin, and a final series was stained with Cresyl violet. All three series were mounted and cleared similarly to that done for the parvalbumin staining procedures described above. Detailed protocols for all stains are freely available (http://lethbridgebraindynamics.com/immunohistochemistry_protocols).

### Image acquisition

Rapid image acquisition of entire coronal or sagittal sections was conducted using NanoZoomer whole-slide scanning microscopy (NanoZoomer Digital Pathology RS, Hamamatsu Photonics), which is capable of automatically capturing wide-field multispectral fluorescent images over entire brain sections at high resolution (Montes-Rodriguez et al., 2013). The objective was focused on the middle of the section in the z dimension and image acquisition was conducted with 40× magnification with a multi band pass filter cube (DAPI/FITC/Texas Red). Following NanoZoomer image acquisition, confocal single plane optical sections from the PC surrounding the injection site were acquired using a FV1000 laser (Olympus, America, Inc.). These confocal images were used to verify the extent of the injection site for each animal administered CTB or FG tracer.

### Cortical flat-maps and automated detection of retrograde tracer

**Figure 2:**
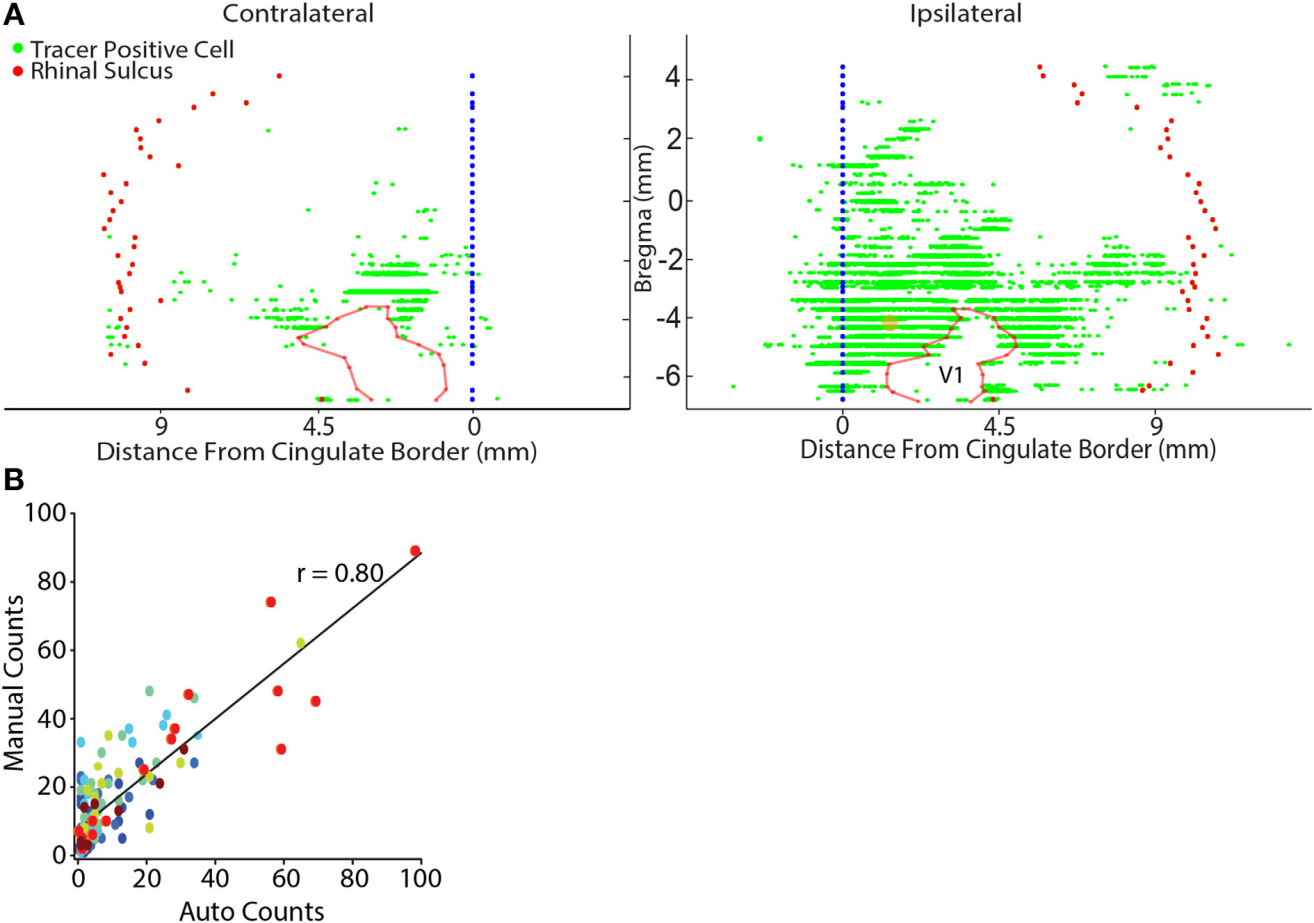
**Representative flat map showing cortical inputs to parietal cortex. (A)** Retrograde tracer positive cells (green dots) were automatically identified (see Materials and Methods) and their position was projected onto a reference line that marked the cortical boundary between layers IV and V. Each reference line was aligned to the lateral border of the cingulate cortex (blue filled circles) and the location of the rhinal sulcus (red filled circles) was marked to provide a lateral cortical reference point. Generally, contralateral labeling was limited to sparse labeling in the lateral entorhinal cortex and homotopic labeling for the tracer injected region. The data shown here was an exception because modest labeling was also observed in contralateral sensory and lateral visual cortices. Note in this example flat-map the general absence of labeled cells in V1 which is outlined with a semi-transparent red line. The center point of the retrograde tracer injection is marked with a semi-transparent orange dot. **(B)** There is a linear relationship between the results of manual counts and automated tracer detection for each rat (each color represents the data for a different rat). Linear regressions were performed on the individual rat data sets and were highly significant in each case (Table 2). Automated counts always underestimated the actual (manual count); therefore, the offset of the linear regression from zero was used to adjust the automated count data on a rat-by-rat basis. A linear regression conducted on the pooled data set was highly significant (*p* < 0.001).

A custom software platform was developed in Matlab (Matlab2013b, Mathworks Inc., Natick, MA) which utilized the open source image processing software, Fiji (Schindelin et al., 2012) (http://fiji.sc/Fiji), to accommodate an automated analysis pipeline generating unfolded 2D cortical maps (Figure 2; Watabe-Uchida et al., 2012). A 2D map was generated for every other NeuN stained section (i.e., evenly spaced sampling of 1/6th of the entire brain). In the case where a section was damaged or other problems prevented automated analysis, the nearest adjacent section was used so that the overall sampling rate remained unchanged. Our aim with these flattened cortical maps was to conveniently illustrate the anatomical position of identified projection neurons from sections processed with NeuN for the entire cortical mantle. First, NanoZoomer images were automatically split into large, but manageable 40× magnification tiles. Second, neurons were automatically identified within each 40× tile using the open source segmentation tool, FARSIGHT (www.farsight-toolkit.org; Bjornsson et al., 2008; Rey-Villamizar et al., 2014). The automated segmentation of neurons allows for measurement of the precise x- y- position of each neuron, as well as the intrinsic neuronal features including the integrated intensity, i.e., the sum of the pixel intensity values within the segmented boundaries of a neuron for each color channel, and the maximum and minimum pixel intensity for each segmented neuron. Based on these measures, we classified neurons as containing retrograde tracer if the total integrated intensity for the appropriate color channel (e.g., red for CTB) exceeded 250,000, and had a maximum pixel intensity exceeding 150 (individual pixel intensity ranged from 0–255). The accuracy of this criterion was validated by correlating estimates of the number of tracer positive cells within a tissue section, with measures obtained from manual counts using the cell counter plugin (http://fiji.sc/Cell_Counter) in Fiji. This analysis yielded a highly significant positive correlation for all comparisons (*p* ≤ 0.01; Figure 2). Following tracer positive cell identification, the position of labeled cells was then mapped onto a reference line manually drawn through the cortical boundary between layers IV and V. The reference line was smoothed using the “runline” function of the Chronux data analysis toolbox in Matlab (www.chronux.org; Mitra and Bokil, 2007), which implements a moving-window line regression with a window size of 5 pixels (1.15 μm) and a step size of 1 pixel (0.23 μm). Each reference line was automatically aligned to the lateral border of the cingulum border and the location of the rhinal sulcus was marked to provide a lateral cortical reference point. When needed, anatomical boundaries were also marked in the same manner as the rhinal sulcus and plotted on the flat map (e.g., see V1 on Figure 2).

**Table 2:** **Linear regression between manual and automatic counts for each rat used for cortical ROI analyses produced the following *r*-values and manual count x-intercept**.

**Rat**	***r*-value**	***p*-value**	**x-intercept**
1	0.78	<0.001	−2.6
2	0.79	<0.001	−1.9
3	0.90	≤0.003	−3.9
4	0.81	<0.001	−4.8
5	0.61	≤0.003	−0.1
6	0.81	<0.001	−0.1
7	0.49	≤0.01	−0.9
8	0.94	<0.001	−3.9
9	0.84	<0.001	−3.3
10	Manual only^a^	Manual only^a^	Manual only^a^

a *Labeling in this rat was sufficiently low intensity to render automated counting unreliable; therefore only data from manual counts was used. For this rat manual counts were sampled at the same interval as automatic counts*.

### Quantification of injection location

The center of the retrograde injection site was defined as a zone in the PC that had a high ratio of labeled fibers with few labeled cell bodies. For anterograde tracer injection site the opposite definition was used: high ratio of labeled cell bodies to labeled fibers. Tracer quantification in the injected region was set to zero. For BDA injected brains, this was verified using NanoZoomer images; however, in fluorescent CTB and FG, the injection region was typically overexposed, leading to a misrepresentation of its actual size. Thus, we verified the extent of the injection site for each CTB and FG injected animal using confocal microscopy (methods described above). Following confirmation of the injection boundaries, estimates of injection size were derived using measurement tools in Fiji. For CTB, the size ranged from 321 to 881 um, for FG the injections ranged from 1400 to 1800 um, and for BDA the injections ranged from 300 to 356 um. The anterior-posterior and medial-lateral location of the injection site was estimated by comparing the section with corresponding plates from Paxinos and Watson (2007). Finally, to quantify the medial-lateral injection placement for each animal, we measured the distance of the central location of the injection site relative to the medial border of the cingulate cortex using our custom matlab software.

### Region of interest analyses

Tracer density was estimated for all areas of the cortex by manually drawing an outline around each region of interest (ROI) using the manual selection tool in Fiji. Once a list of ROIs with tracer was obtained, only the blue (NeuN) channel was selected so the ROI boundaries could be drawn blind to tracer. Cortical ROIs were based on regional boundaries provided by Paxinos and Watson (2007) and cytoarchitectural differences observed in adjacent series of NeuN, Cresyl violet, and parvalbumin stained tissue. These boundaries are readily identifiable in NeuN-stained sections, especially when aided with adjacent parvalbumin and Cresyl stained sections, using standard cytoarchitectural criteria such as cell packing densities and thicknesses of layers. The number of labeled cells was then calculated for each cortical zone. For each animal, an additional set of manual counts were obtained for each ROI. These manual counts were acquired only for a subset of sections by starting with every fourth automatically quantified section and incrementally increasing the sampling rate until at least 10–15 regional measurements were obtained for the animal. The full data set of automated cell counts was adjusted based on the offset of the x-intercept of the regression line for manual vs. automated counts. In every case this offset was negative, so a value ranging from 0.1 to 4.8 was added to each cortical ROI automated tracer positive cell count. We used two dependent variables for group and region comparisons. First, the raw total number of sections was used. Second, group totals were counted and normalized to the proportion of total counts. The ROI (or two in the case of injections that straddled borders) which included the center of the injection site (as defined using confocal microscopy, see above) was set to zero for all sections. Labeled neurons in the thalamus were counted manually rather than with automated methods due to the difficulty of accurately segmenting the neurons observed throughout this region. Thalamic ROIs were again based on regional differences in cytoarchitecture. Cortical and thalamic ROIs were referenced from Paxinos and Watson (2007).

### Statistics

Measures of the proportion of tracer in cortical and thalamic regions was subjected to one-way repeated measures analysis of variance (RMANOVA) and comparisons between groups of animals with injections in rostral vs. caudal and medial vs. lateral locations were subjected to two-way repeated measures RMANOVAs. Bonferonni *post-hoc* tests were used to evaluate differences between individual ROIs. Results were considered significant for *p* < 0.05.

## Results

### Low-density primary visual cortex projections to parietal cortex

Whole brain cortical flat maps showing retrograde tracer filled cells were generated for each brain that was sectioned in the coronal plane (*n* = 10). Based on our initial inspection of each flat-map, we observed that despite inclusion of a “visual” area often characterized as V2MM and V2ML (Paxinos and Watson, 2007) in our definition of PC, the density of inputs from primary visual cortex (V1) were in fact quite low. This is particularly apparent on most flat-maps by the absence of retrogradely labeled cells in this zone (see Figure 2).

### High-density retrosplenial cortex inputs to parietal cortex

To quantify the relative density of retrogradely labeled neurons in regions sending output to PC, we calculated the projection density from each cortical ROI based on the automated tracer positive cell counts. We did this in two ways. First, we calculated the total number of tracer positive cells. Since we sampled 1 in 6 sections through the whole brain, the number of cells reported here would be roughly equivalent to 1/6th of the total number of labeled cells. Next, we calculated the proportion of the total labeled cells for each region. Consistent with previous reports, retrogradely labeled cells were identified in medial prefrontal, motor, somatosensory, auditory, lateral visual and retrosplenial cortical areas (Kolb and Walkey, 1987; Reep et al., 1994) and were almost exclusively unilateral in nearly every case, except for homotopic labeling in the contralateral hemisphere (Figure 2). Therefore, for subsequent analyses we only included the brain hemisphere ipsilateral to the injection and only included regions that had readily identifiable projections to the PC. In general, the PC receives input from each of the four networks defined by Zingg et al. (2014): medial, lateral, somatic, and claustrum/entorhinal cortex. The strongest labeling was observed in the medial group, consistent with PC membership in this network. This observation was supported by significant variations in the proportion of labeled cells across the four networks (one-way RMANOVA *F*_(3, 39)_ = 24.16, *p* < 0.001). Further, inputs to PC from the medial network were significantly greater than the remaining three networks (Bonferonni corrected post-test; *ps < 0.001*) which didn't differ from each other. Identical results were obtained for the total number of sampled cells (one-way RMANOVA *F*_(3, 39)_ = 7.90, *p* < 0.001; post-test *p*s < 0.05).

**Figure 3:**
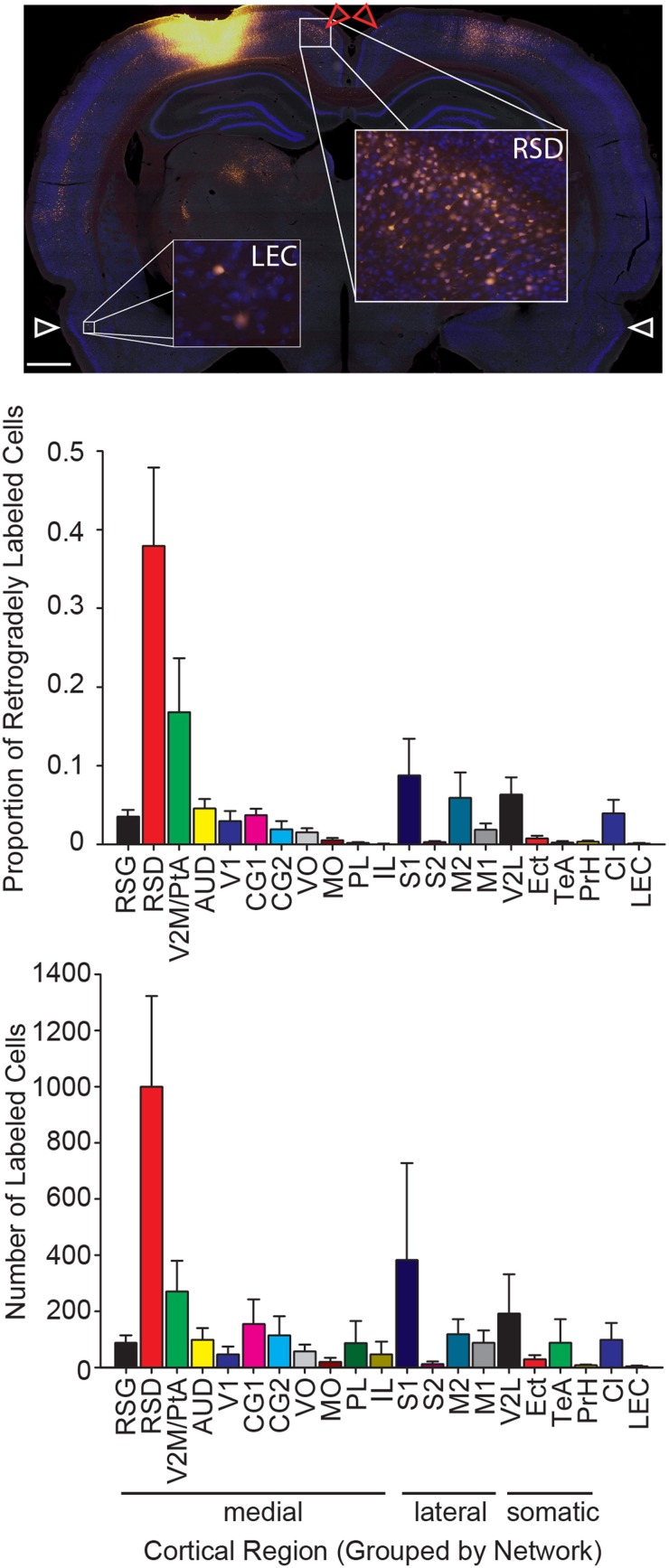
**The strongest input to parietal cortex emerges from dorsal retrosplenial cortex. Top:** Representative image of a brain section at the level of the dorsal retrosplenial cortex and lateral entorhinal cortex from a rat with a Fluoro-Gold injection. Note that a greater number of Fluoro-Gold labeled cells (yellow filled cells) is present in the dorsal retrosplenial cortex (RSD; red arrows) compared to the lateral entorhinal cortex (LEC; white arrows). Scale bar = 1 mm. **Middle:** The proportion of retrogradely labeled neurons within each region of interest (Mean ± s.e.m.). **Bottom:** The number of retrogradely labeled neurons for each region of interest in each cortical network (Mean ± s.e.m.). Note that the projection density to parietal cortex is greatest from the medial network, particularly the dorsal retrosplenial cortex.

More specifically there was significant variation in the proportion of tracer labeled cells across all 21 cortical regions that had readily identifiable projections to the PC (one-way RMANOVA *F*_(20, 208)_ = 7.83, *p* < 0.001). The largest input to PC from within the medial network originates from the dorsal retrosplenial cortex (RSD; Figure 3), which is also referred to as dysgranular retrosplenial cortex or Brodmann's area 30 (Wyss and Van Groen, 1992; Sugar et al., 2011). This observation was also supported by post-testing, which showed that the density of projections from dorsal retrosplenial cortex to PC was significantly greater than from any other region within the medial network (*p*s < 0.001), which did not differ from each other. In addition, the average projections to PC from regions other than the injected region (i.e., intrinsic inputs) were significantly greater than mPFC, Te, and LEC projections to PC (*p*s < 0.05). Almost identical results were obtained for the total number of sampled cells (one-way RMANOVA *F*_(20, 208)_ = 3.50, *p* < 0.001; retrosplenial projections to PC were greater than any other region, *p*s < 0.05); therefore, for the remaining cortical analyses, only the proportion of labeled cells was used.

### Low-density inputs from the entorhinal cortices to the parietal cortex

**Figure 4:**
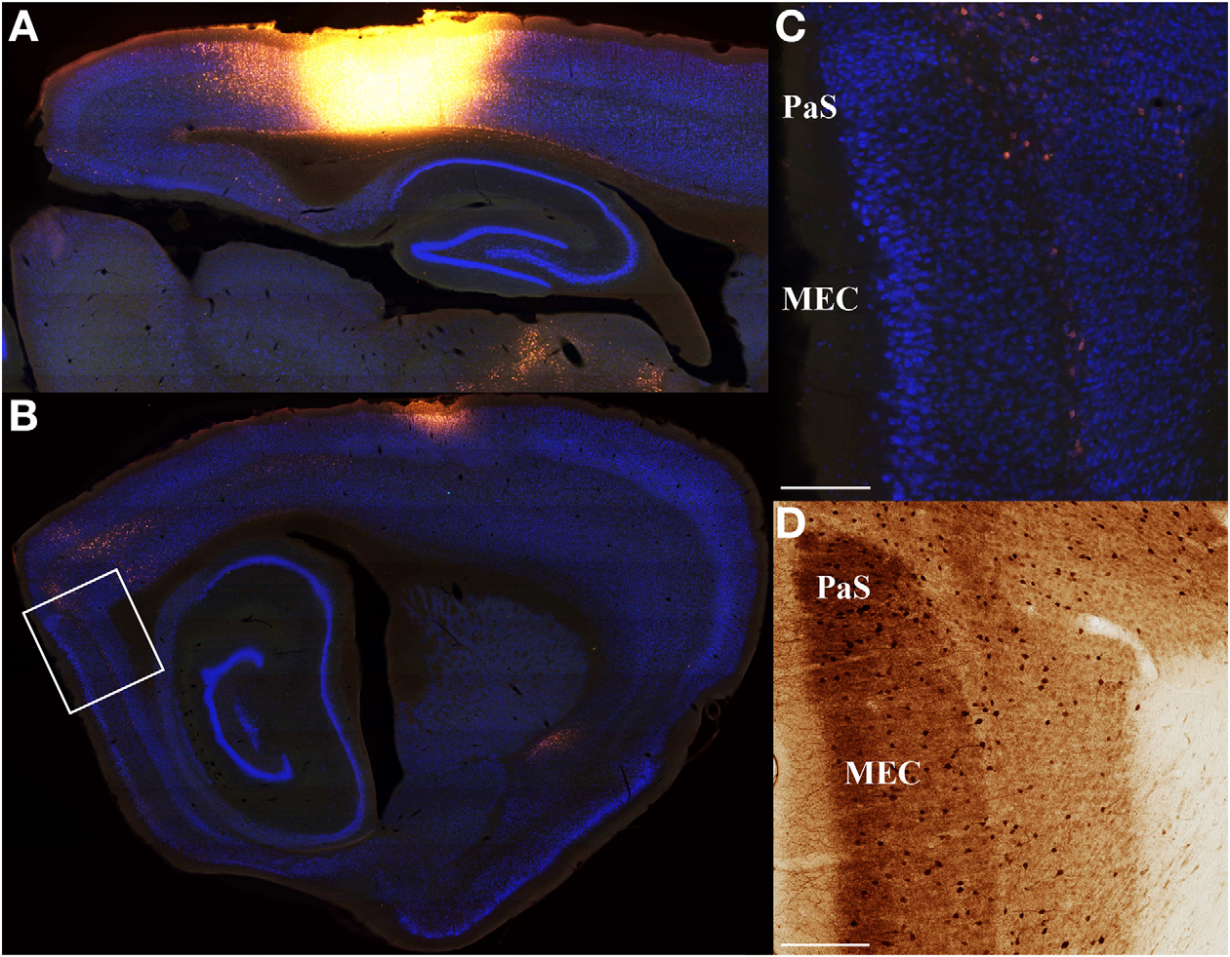
**Minimal input from entorhinal cortex to parietal cortex**. Saggital section retrograde tracer data from three rats was used to confirm that entorhinal cortex inputs to parietal cortex are weak. An example is shown for a very large parietal cortex injection of Fluoro-Gold **(A)** that produces very weak labeling in medial entorhinal cortex (MEC; **B**). **(C)** High magnification digital micrographs confirm the weak labeling shown in **(B)**. As with coronal sections, adjacent parvalbumin stained sections were used to define regions of interest and differentiate from adjacent structures. Similar results were obtained for projections from parietal cortex to entorhinal cortex (i.e., anterograde; **Figure 10**). Though medial entorhinal cortex labeling is sparse, it is highly specific. Projections from medial entorhinal cortex (MEC) to parietal cortex arise almost exclusively from the layer IV\V border. **(D)** Adjacent parvalbumin stained section used to assist in identifying the regions shown in **(C)**. The location of the high magnification digital micrographs shown in **(C,D)** are indicated with a white box in **(B)**. Parasubiculum (PaS). Scale bars = 250 μm.

**Figure 5:**
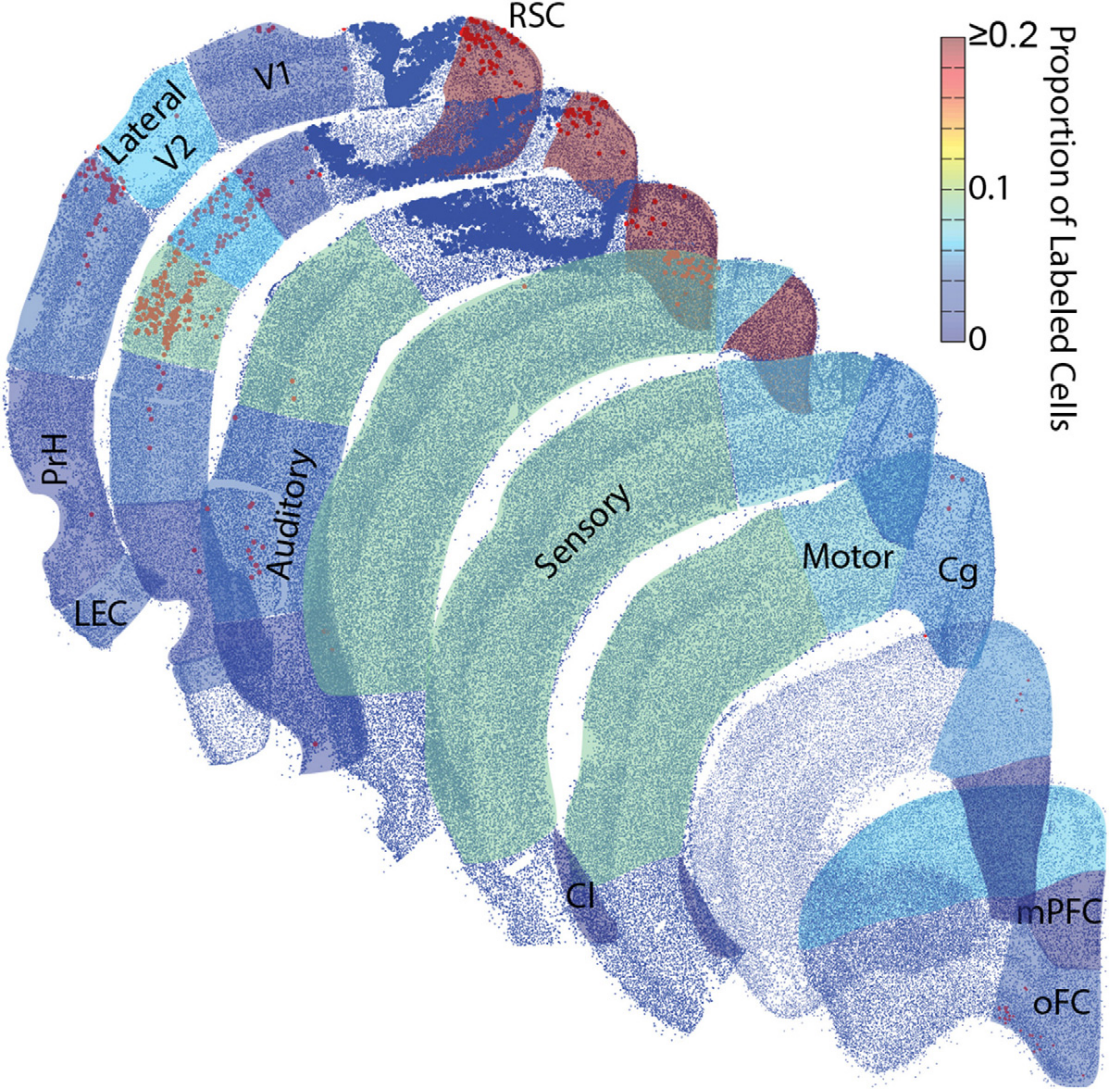
**Rat parietal cortex receives a majority of inputs from the medial network**. Representative sections were reconstructed by plotting the center point of each identified cell (small blue dots) and tracer positive cell (red/orange dots). Then collapsed regions of interest were shaded with 50% transparency with a colormap for the average proportion of retrogradely labeled cells identified there across all rats. These dot plots are surprisingly realistic. Even though single dots and not actual tissue is shown, the packing density of these dots produces identifiable borders between cortical layers. Each region with identifiable tracer positive cells was shaded, unshaded regions did not have identifiable projections to the parietal cortex. Note, most of the inputs to the parietal cortex arise from the network to which it belongs, the medial network, particularly from a single structure within the medial network, the retrosplenial cortex. Larger dark blue dots were in the same parietal cortex region(s) as the injection and were excluded. Retrosplenial cortex (RSC) includes RSD and RSG. Medial Prefrontal cortex (mPFC). Orbital Frontal cortex (oFC) includes LO, VO, and MO. Cingulate cortex (Cg) includes Cg1 and Cg2. Clastrum (Cl). Lateral Entorhinal cortex (LEC). Perirhinal cortices (PrH) includes PrH, Ect and Te.

A majority of our data was collected in the coronal plane, which often prevented collection and processing of the most posterior-medial extent of cortex, especially parahippocampal structures such as the medial entorhinal cortex and parasubiculum. Thus, to determine whether these areas send projections to the PC, we collected data in the sagittal plane in a subset of animals (*n* = 3). Visual inspection of these data revealed that projections from the medial entorhinal cortex were generally weak (Figure 4) and were comparable to projections originating from the lateral entorhinal cortex described above. Though projection density to the PC was low it was interesting that essentially all of the retrogradely labeled cells in the EC were localized to layer V along the boundary with layer IV. This layer specificity was consistent with previous descriptions of medial entorhinal cortex outputs to isocortex (Swanson and Kohler, 1986). We also found little evidence of retrograde labeling in the parasubiculum or presubiculum (Figure 4), suggesting that the parahippocampal cortex provides little direct input to the PC. Thus, retrosplenial cortex appears to serve as an interface between parietal cortex and the parahippocampal region (Summarized in Figure 5).

### Cortical projections to rostral and caudal parietal cortex are similar

**Figure 6:**
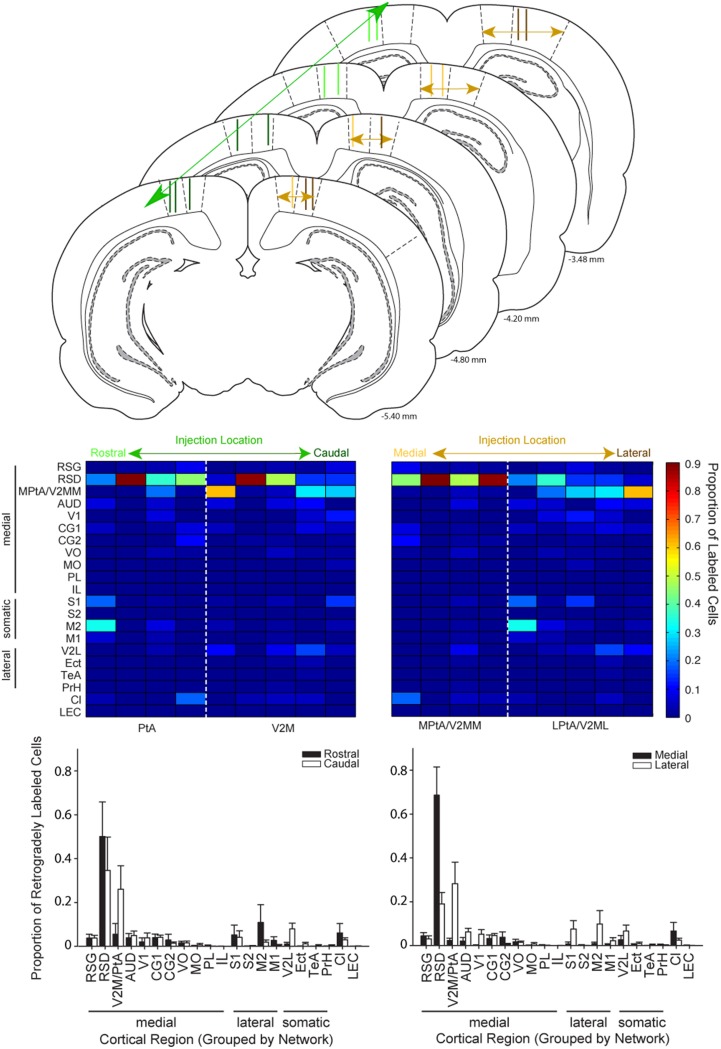
**Cortical inputs to parietal cortex vary as a function of injection location along the medial-lateral but not rostral-caudal axis. Top:** Schematic illustrating the range of injection site locations within parietal cortex. Injection tracts for each rat used in this analysis are shown two times, one time on each hemisphere. Injections of retrograde tracer in parietal cortex were classified along the rostral-to-caudal axis (left column) or the medial to lateral axis (right column). Schematics are adapted from Paxinos and Watson (2007). Distance from bregma is indicated in the bottom right corner for each schematic. **Middle:** Colormaps were generated from the proportion of retrogradely labeled cells for cortical regions (rows) for each rat (columns). Data in the left panel is sorted from left to right according to the rostral to caudal coordinate of the injection site. Data in the right panel is sorted from left to right according to the medial to lateral coordinate of the injection site. Note that labeling varies systematically along the medial-lateral axis but not the rostral-caudal. **Bottom:** Bar plots showing the proportion of retrogradely labeled cells for regions in animals with injections in rostral vs. caudal parietal cortex (left panel) and medial vs. lateral parietal cortex (right panel). The split medial vs. lateral and rostral vs. caudal grouping was made based on anatomical boundaries. For example, PtA centered injections were classified as rostral and V2M centered injections were classified as caudal. The population data shown here confirmed the observations from the colormaps. Splitting the data for these groups in a variety of ways produced the same result (see text). There was a significant interaction between region for medial vs. lateral comparisons (two-way RMANOVA; *F*_(20, 140)_ = 3.85, *p* < 0.0001), but not anterior vs. posterior comparisons (two-way RMANOVA: *F*_(20, 140)_ = 1.01, *n*s). V2M/PtA refers to any remaining V2M and PtA regions that were not part of the tracer injection site. Each injection spanned 1–2 regions so this encompassed the 2–3 remaining regions.

Given that the parietal area of isocortex is often described as containing 2 or 4 distinct regions (Burwell and Amaral, 1998; Paxinos and Watson, 1998, 2007; Whitlock et al., 2008) we examined the possibility of regional variation with respect to the rostral-caudal location of retrograde tracer injection into the PC. To illustrate potential differences, ROI data for each individual animal were plotted as colormaps and ranked according to the center of their rostral-caudal injection location. Injection center points were determined using a confocal microscope to classify the range of the injection site in the anterior to posterior plane, as described in the methods. Next, two raters (blind to the data) classified the center of the injection site relative to bregma. Then, the animals were sorted according to corresponding bregma value. Note, one animal was excluded from these analyses because the injection spread was very large and covered all four regions targeted in the present study (*n* = 9 rats). Visual inspection of the sorted data from all animals did not reveal prominent differences in the rostral to caudal plane (Figure 6 Left Column). This was confirmed by a lack of interaction between region and group (rostral vs. caudal injection), when the data were split based on anatomical boundaries (two-way RMANOVA: *F*_(20, 140)_ = 1.01, *n*s; Paxinos and Watson, 2007). We also conducted the same analysis and found no difference after splitting the data in “half” a different way (*n* = 5 rostral and 4 caudal; *F*_(20, 140)_ = 0.11, *ns*) vs. in “half” (*n* = 4 rostral and 5 caudal) as was done using the anatomical boundary above. In other words we tried moving the boundary between rostral and caudal back just enough to include one more injection in the rostral group and this did not change the result.

### Distinct cortical projections to medial vs. lateral parietal cortex

Injections placed in medial vs. lateral regions of PC were then compared. Two experimenters (blind to the data) classified the relative medial-lateral injection position similar to the procedures described above. Next, we generated a heat map with animals sorted according to the medial to lateral position of their PC injection (Figure 6 Right Column). Then, ROI data was split into two groups based on the medial vs. lateral anatomical border (Paxinos and Watson, 2007). Visual inspection of this data revealed a shift in projection patterns from medial vs. lateral PC injections, particularly when low density projections were re-scaled (Supplementary Figure 1). This observation was confirmed by a significant interaction between group (medial or lateral) and brain region (two-way RMANOVA; *F*_(20, 140)_ = 3.85, *p* < 0.0001). As an additional test we used the injection point centers to compute the distance of the injection site from the cingulum border (see flat-map and injection center methods above). Although this method does not account for the PC narrowing in caudal regions, it produced a similar pattern of results as the rater method above, as indicated by a significant interaction between group and brain region (*F*_(20, 140)_ = 3.43, *p* < 0.0001). Finally, the two most medial injections based on rater classifications were excluded to ensure injection spread into the retrosplenial cortex was not responsible for the effects we observed. Again there was a significant interaction between group and brain region (*F*_(20, 115)_ = 3.19, *p* < 0.0001).

### Distinct thalamic projections to medial and lateral parietal cortex

**Figure 7:**
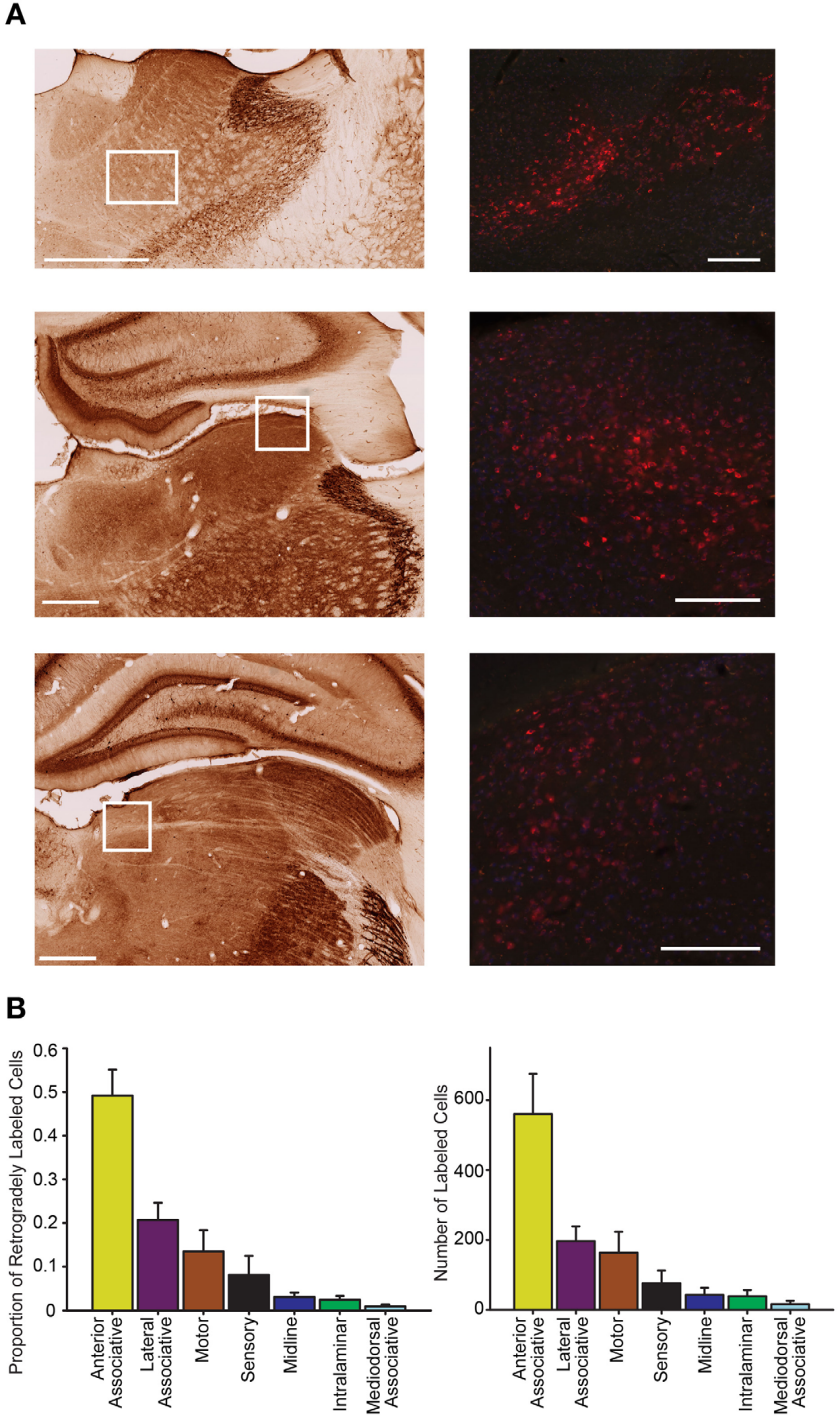
**Parietal cortex receives dense inputs from associative thalamus**. **(A)** Representative labeling is shown in the anteromedial and anteroventral thalamus (top) in an animal injected with CTB (red = CTB). These thalamic nuclei are part of the anterior associative group. The left panel shows the regional boundaries in the adjacent parvalbumin stained section. Middle representative labeling is shown in the lateraldorsal thalamus, part of the lateral associative group. Bottom representative labeling is shown in the lateral posterior medial thalamus, part of the lateral association group. The approximate location of each higher magnification image of CTB labeled cells is marked with a white box. Scale bars = 500 μm. **(B)** The proportion of retrogradely labeled cells varied significantly across the thalamic zones (Left; one-way RMANOVA *F*_(6, 69)_ = 18.07, *p* < 0.001). Note the significantly higher density of projections to parietal cortex from anterior associative group. Right Very similar labeling patterns to those seen with the proportion of labeled cells are observed when the number of retrogradely labeled cells is compared across regions (*F*_(6, 69)_ = 13.61, *p* < 0.001).

Consistent with previous reports (Kolb and Walkey, 1987; Reep et al., 1994; Kamishina et al., 2009), our PC injections resulted in abundant retrograde labeling in the thalamus (Figure 7). Labeled neurons were almost exclusively restricted ipsilateral to the injection site and largely spanned across 5 thalamic groupings defined by Groenewegen and Witter (2004): motor, sensory, associative (anterior, lateral, mediodorsal), intralaminar, and midline nuclei. In general, however, the strongest labeling was observed in lateral and anterior associative nuclei and moderate labeling in sensorimotor thalamic groups (Figure 7). This observation is supported by a significant variation in the proportion of projecting cells across each thalamic group (one-way RMANOVA *F*_(6, 69)_ = 18.07, *p* < 0.001), and the significantly higher means for anterior and lateral associative nuclei relative to the weak labeling observed in midline and intralaminar nuclei (all comparisons *p* < 0.05). Similarly, the number of cells significantly varied across each thalamic group (one-way RMANOVA *F*_(6, 69)_ = 13.61, *p* < 0.001) and was higher for anterior associative nuclei relative to all other groups (*p* < 0.001). Given the high degree of similarity of numbers of cells and proportions of cells and the reduction in variability that accompanies the proportion measure, further analyses on thalamic data were conducted on the proportion of cells only. In sensory and motor groups, a moderate number of tracer positive neurons were identified in all ventral and posterior thalamic nuclei (Figure 7). In the anterior associative group, a greater proportion of labeled cells was observed in the anteroventral and anteromedial thalamus, and little to no labeling was observed in the anterodorsal thalamus. Projections from lateral associative nuclei were largely restricted to the laterodorsal thalamic nuclei and the lateroposterior medial-rostral nuclei, but there was little labeling observed in the lateroposterior lateral-rostral nuclei.

**Figure 8:**
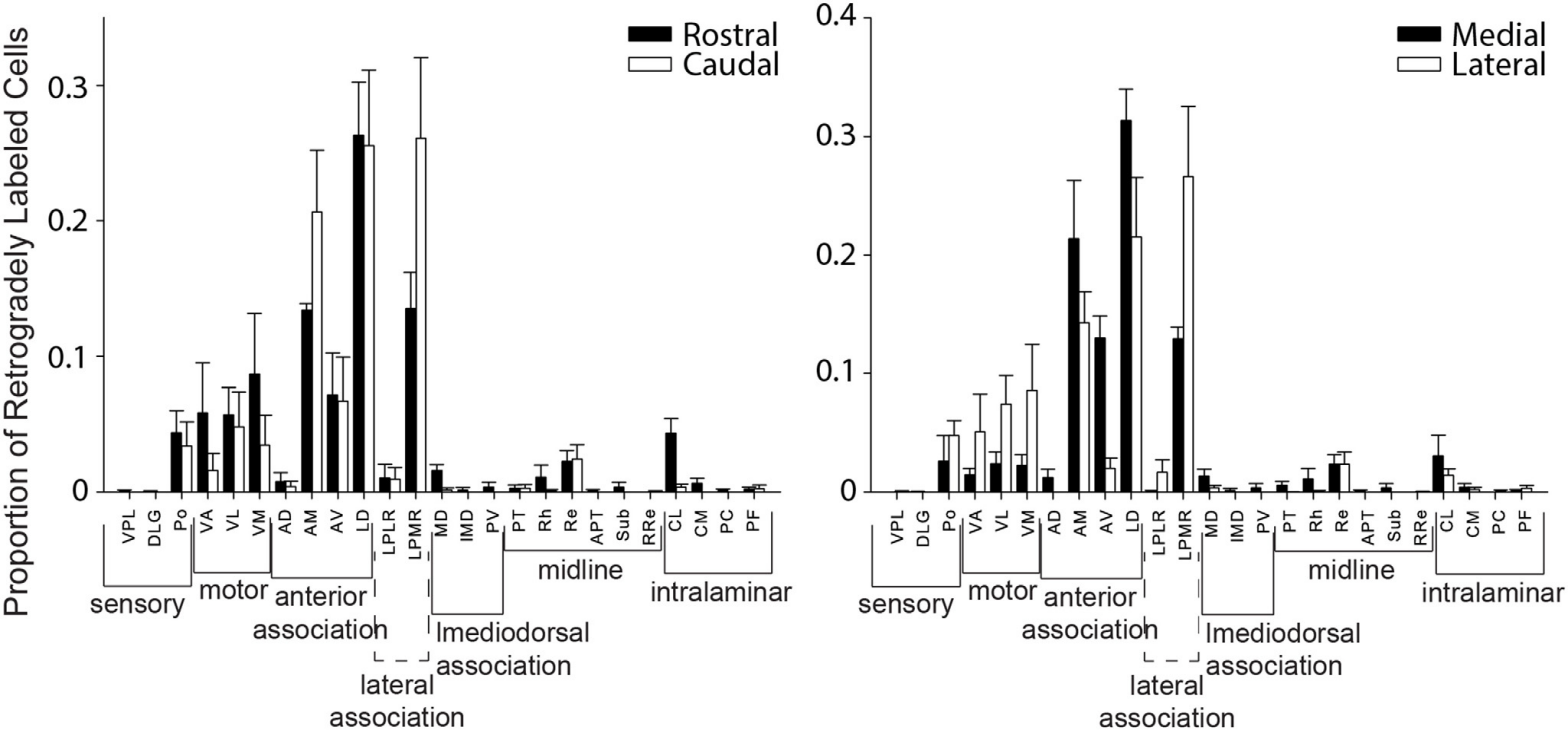
**Distinct thalamic projection patterns to medial vs. lateral but not rostral vs. caudal parietal cortex**. Plots showing the proportion of retrogradely labeled cells for each region within thalamic groups for injection in rostral vs. caudal (**left panel**) and medial vs. lateral (**right panel**) parietal cortex. A significant interaction was observed between group and region for thalamic projections to medial vs. lateral parietal cortex (two-way RMANOVA; *F*_(24, 168)_ = 3.22, *p* < 0.0001), but not rostral vs. caudal parietal cortex (*n*s). This thalamic projection pattern mirrors that seen in cortical projection patterns to parietal cortex (see Figure 5). Interestingly, in both thalamus and cortex there are more motor inputs to lateral parietal cortex.

**Figure 9:**
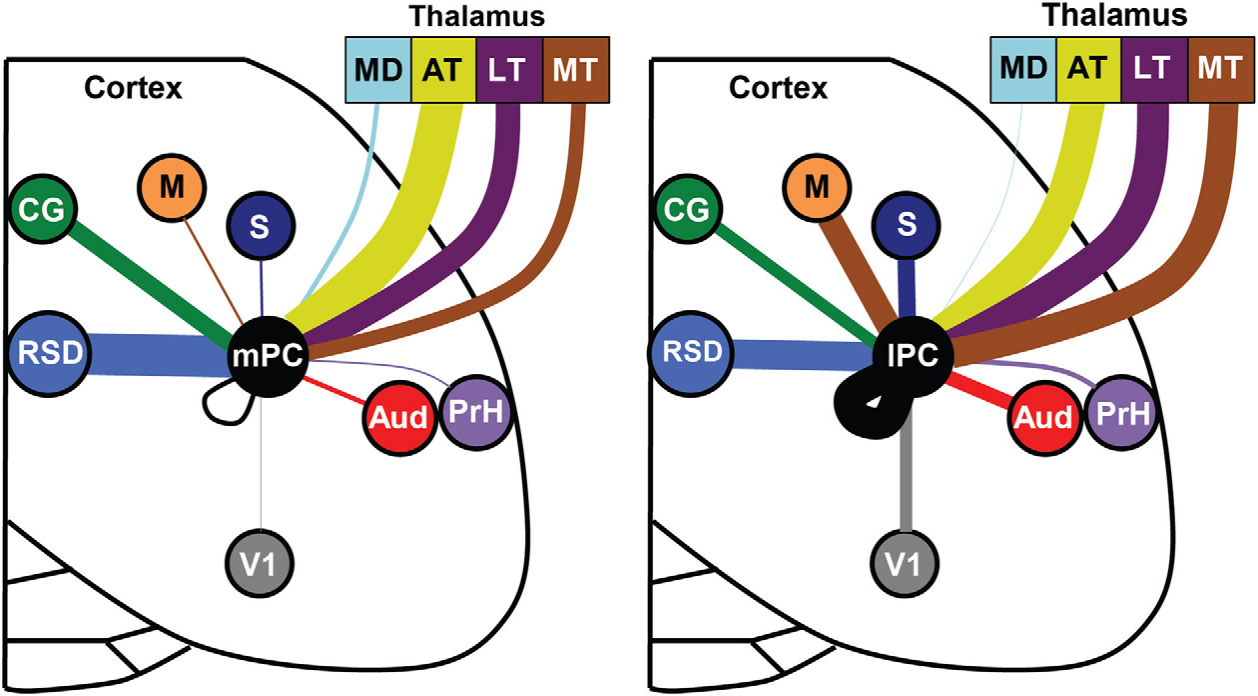
**Distinct input patterns for medial vs. lateral parietal cortex**. Each brain region is indicated by a different color and the line thickness represents the strength of thalamic and cortical projections to the medial PC (**left panel**) and lateral PC (**right panel**). Only regions with significant medial vs. lateral differences in projection strength are shown (i.e., non-overlapping error bars). Thalamic data was normalized separately from cortical data. Note that projections to lateral PC (lPC) involve stronger projections from the somatosensory (S), motor (M), visual (V1), auditory cortex (AUD), and motor thalamus (MT). The medial PC (mPC) receives stronger inputs from the dorsal retrosplenial cortex (RSD) and cingulate region (CG). Mediodorsal thalamus (MD), anterior thalamus (AT), lateral thalamus (LT), perirhinal cortex (PrH).

We also evaluated whether thalamic projections differentially target distinct rostral-caudal (i.e., MPtA/LPtA vs. V2MM/V2ML) or medial-lateral (i.e., MPtA/V2MM vs. LPtA/V2ML) zones of the PC. Consistent with cortical input pattern, there was a significant interaction between medial/lateral group and brain region (two-way RMANOVA; *F*_(24, 168)_ = 3.22, *p* < 0.0001), but not rostral/caudal group and brain region (*F*_(24, 168)_ = 0.15, *n*s). Animals with injections restricted to the MPtA/LPtA displayed greater thalamic inputs from nuclei in the sensory and motor groups compared to injections targeting V2MM/V2ML (Figure 8). In contrast, animals with posterior injections displayed a greater proportion of inputs from the laterodorsal thalamus, and the proportion of projections from motor and sensory thalamic nuclei was significantly lower relative to the anterior PC. Inputs from the anterior thalamus did not vary as a function of anterior-posterior injection locus, but did vary in relation to the medial-lateral injection location. Specifically, medial portions of the PC received a higher proportion of anteroventral and anteromedial thalamic input. Furthermore, laterodorsal thalamus projected more heavily to the medial PC while the lateroposterior medial-rostral nucleus of the thalamus projected more heavily to the lateral PC. In addition, a greater proportion of motor nuclei projected to the lateral PC. The weak labeling observed in mediodorsal associative, midline, and intralaminar thalamic nuclei did not vary appreciably with anterior-posterior or medial-lateral injection location. In sum, the thalamus and cortex form distinct projection profiles to medial and lateral PC (Figure 9).

### Parietal cortex projects strongly to retrosplenial cortex

**Figure 10:**
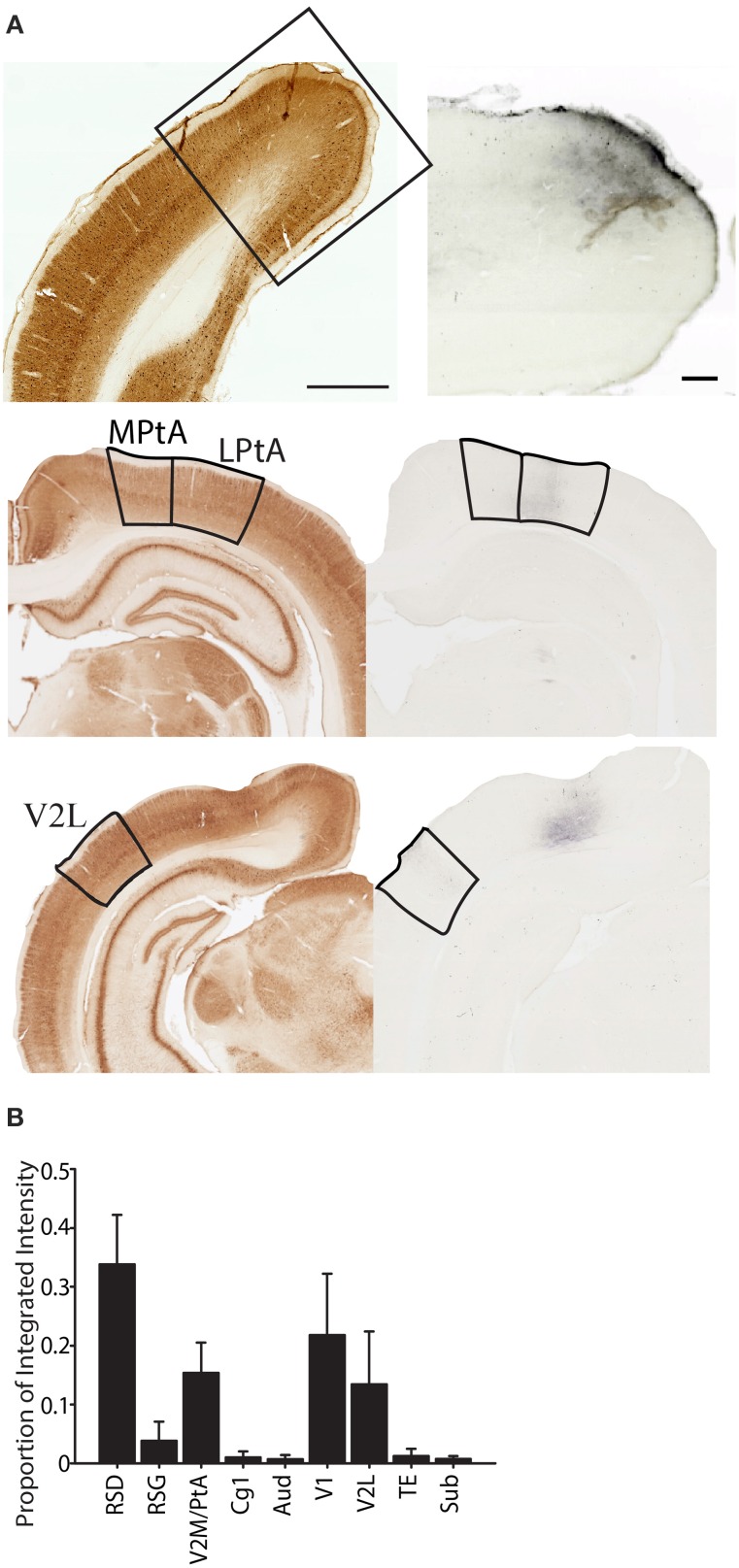
**Projections from parietal cortex terminate predominately in dorsal retrosplenial cortex. (A)** Left Parvalbumin stained section that was used to identify the region where the terminal labeling was observed. When necessary the adjacent section was aligned and overlaid in ImageJ to differentiate regions. Right terminal labeling in dorsal retrosplenial cortex (RSD; top), Medial and lateral Parietal Association Cortex (MPtA; LPtA; middle) or lateral secondary visual cortex (V2L; bottom) following anterograde transport of high molecular weight biotinylated-dextran-amine (BDA) that was injected into the parietal cortex. **(B)** Proportion of total integrated intensity for each region with terminal labeling following parietal cortex injections (Mean +/− s.e.m.). Projections from parietal cortex are mostly within the medial network to which parietal cortex belongs and a majority of projections are to the dorsal retrosplenial cortex (RSD; *p* < 0.05). V2M/PtA refers to any remaining V2M and PtA regions that were not part of the tracer injection site. Each injection spanned 1–2 regions so this “region” encompassed the 2–3 remaining regions. Parvalbumin scale bar = 1 mm; BDA Scale bar = 250 μm.

Finally, given that several recent recording studies have been directed toward understanding the contributions of PC to the spatial functions of the entorhinal cortex and hippocampus, we also examined the major cortical outputs of the PC using the high molecular weight anterograde tracer BDA (*n* = 4; Figure 10). Overall, we found that PC outputs were similar to cortical inputs with a majority of the projections terminating in dorsal retrosplenial cortex and lower density projections to other PC subregions (e.g., LPtA to V2ML), cingulate cortex (Cg1), temporal cortex (TE), lateral secondary visual cortex (V2L) and primary visual cortex (V1). Consistent with this observation, there was a significant variation in projection density across brain region (one-way RMANOVA: *F*_(9, 39)_ = 4.08, *p* < 0.01) and projection density to dorsal retrosplenial cortex (RSD) was significantly greater than all regions (Bonferroni corrected *post-hoc* testing *p*s < 0.05) except other PC regions, V1, and V2L, which did not differ from each other (*n*s). This suggests that the function of PC is tightly coupled to dorsal retrosplenial cortex, since bidirectional connectivity between restroplenial cortex and PC is very strong. It is also interesting to note the PC output to visual cortices (V1 and V2L) is also relatively strong, suggesting PC may be part of a circuit providing significant feedback to these regions. Finally, it is interesting to note that subjectively injections of anterograde tracer into more medial parietal regions produced stronger dorsal retrosplenial cortex labeling and weaker primary and secondary lateral visual cortex labeling. However, anterograde tracer injections into more lateral parietal regions produced stronger primary and secondary visual cortex labeling but weaker retrosplenial labeling.

## Discussion

Since we observed functionally similar cell types in rodent medial secondary visual cortex (V2MM/V2ML) to those observed in primate posterior PC (Wilber et al., 2014) we set out to map this relatively uncharacterized region and compare it to rostral (parietal association: Pta) cortex. The results show that the connectivity pattern of rat medial secondary visual cortex and parietal association cortex are remarkably similar (i.e., there was no evidence for robust connectivity differences along the rostral-caudal axis of the PC). However, we found that the lateral portions of PC have a connectivity profile that is distinct from the medial zones. Specifically, medial portions were almost exclusively reciprocally connected to dorsal retrosplenial cortex, and received dense inputs from anterior and lateral associative thalamus. While the lateral portion received inputs from a broader range of cortical and thalamic structures including medial secondary visual and parietal, motor, sensory and visual cortices, sensory and motor thalamus. Interestingly, rat caudal PC received, at most, a low-density primary visual cortex input.

The pattern of labeling we observed in rostral PC (i.e., “PtA”) is consistent with previous reports in rats (Kolb and Walkey, 1987; Reep et al., 1994) and mice (Lim et al., 2012; Wang et al., 2012; Oh et al., 2014; Zingg et al., 2014). Specifically, we found that PC receives inputs from a broad range of cortical and thalamic regions including the medial prefrontal, motor, somatosensory, auditory, lateral visual, primary visual, cingulate and retrosplenial cortical areas and motor, sensory, associative, intralaminar and midline thalamic nuclei. Rodent anatomical studies have excluded medial PC regions (e.g., MM in the mouse) or collapsed across medial and lateral regions (e.g., single PtA not LPtA and MPtA). Thus, our results provide the first insight into anatomical distinctions between medial and lateral PC in rodents.

With the exception of one study characterizing projections from the thalamus to V2M (Kamishina et al., 2009), the cortical and thalamic inputs and outputs to the posterior region of PC had not received similar attention in rats. However, recent studies have characterized the lateral (though not medial) portion of this posterior regions in the mouse, the anterior-medial and posterior-medial cortex (AM and PM), and our results suggest that these lateral posterior regions AM/PM have similar connectivity to the posterior-lateral region of rat PC (i.e., V2ML). For example, in both rodents these analogous regions are heavily connected (reciprocally) with the retrosplenial cortex, and with a relatively low-density to the entorhinal cortex. Mouse AM/PM and rat V2ML both show similar inputs, including the retrosplenial, cingulate, sensory, motor, and orbital cortex (Wang et al., 2012; Oh et al., 2014; Zingg et al., 2014). However, our data is not entirely consistent with mouse studies, which generally suggest stronger V1 projections to posterior PC (i.e., AM/PM) than we reported in rats, suggesting a possible species difference (Wang et al., 2012; Oh et al., 2014; but see: Zingg et al., 2014). It is also possible that a methodological difference actually explains this discrepancy because manual counts using an anterograde and retrograde approach suggest lower density V1 inputs to AM/PM (Zingg et al., 2014), while automated anterograde methods suggest higher density V1 inputs (Wang et al., 2012; Oh et al., 2014). In addition, PC is known to contain axons from V1 projecting to frontal cortex, which could produce false positives when using automated techniques for quantifying anterogradely labeled fibers.

The absence of distinct rostral-caudal input patterns observed in the present study is surprising given the range of functional observations observed along this axis (Nakamura, 1999; Nitz, 2012; Whitlock et al., 2012; Wilber et al., 2014). In fact, distinctions in cytoarchitecture suggest that there should be anatomical segregation of such functions along the rostral-caudal axis. While this may be the case, the results of the present study indicate that there is very little variability in anatomical inputs to the rat PtA and V2M. It should be noted that some subtle rostral-caudal differences were observed. For example, laterodorsal thalamus, which receives dense visual inputs, projects more heavily to caudal PC. This specific difference could have functional implications. For instance, visually responsive cells (Kaufman et al., 2013; Wilber et al., 2014), including visually modulated head direction cells (Mizumori and Williams, 1993), may be found with greater frequency in caudal PC.

The significant variability across rats in our study was largely explained by the medial vs. lateral position of retrograde tracer injections. This finding suggests at least two major projection pathways of cortical information to the PC: a distinct projection to the medial PC with a major contribution from the retrosplenial cortex, and a distinct pathway to the lateral PC with a moderate contribution from the retrosplenial cortex, and with higher intrinsic PC connectivity. Projections to lateral PC also had a relatively higher density of inputs from motor, sensory and visual cortices. Interestingly, Reep and Corwin (2009) found evidence of weak functional specialization for attention in medial vs. lateral portions of rat PC, and suggested that anatomical studies should be conducted to compare the connectivity of these sub-regions. Further, functional imaging studies in humans have suggested a similar functional distinction with a medial parietal region for spatial attention and a lateral network that has been implicated in neglect (Corbetta et al., 2008; Howard et al., 2013; Schindler and Bartels, 2013). Wilber et al. (2014) recorded PC neurons across the medial-lateral axis, however, fewer neurons were recorded in medial PC, so it is unclear whether functional differences are expressed along this axis. Similarly, other recording studies in PC have not provided sufficient anatomical information to determine whether route-centric, egocentric, or self-motion signals vary as a function of medial vs. lateral PC (Nitz, 2012; Whitlock et al., 2012). Furthermore, anatomical studies in the mouse have generally neglected to describe the input-output relationships of the medial portion of PC (i.e., MM in the mouse). In an experiment using 2-Photon imaging of the mouse PC, the absence of functional segregation was reported, however it is not clear whether these observations also included comparisons of medial and lateral mouse PC (Harvey et al., 2012).

One of the most surprising findings in the present study was the relatively weak primary visual cortex input to the PC, especially to the medial secondary visual cortical (caudal) zone of PC (i.e., V2MM/V2ML). Previous studies of caudal PC were generally qualitative, and therefore the relative density of V1 inputs was not assessed. Regardless, the strong visual responses of cells in caudal PC begs the question of where visual information conveyed to this region may originate? One possibility is the dorsal retrosplenial cortex, which has been shown in rodents and humans to process visual-spatial information (Vann et al., 2009; Clark et al., 2010; Bucci and Robinson, 2014). A second possibility is that visual input may be conveyed by laterodorsal thalamic regions which rests along the tecto-pulvinar pathway (Thompson and Robertson, 1987) and projects to the PC (Van Groen and Wyss, 1992 and Figure 7). Third, the bulk of the relatively weak visual inputs from V1 and V2L project most heavily to lateral PC, so it is possible that visually responsive cells are largely confined to that region. Moreover, the sparsity of visual cortical inputs may not be a significant issue, for instance, visual inputs from V1 and V2L are comparable to the density of auditory inputs and auditory responses have been observed in rat PC (Nakamura, 1999).

Recent work has focused on the relationship between the PC and brain regions involved in spatial computation, especially the entorhinal cortex (Whitlock et al., 2008, 2010, 2012; Whitlock, 2014) and hippocampus (Wilber et al., 2014). While some earlier studies suggested there may be a direct anatomical pathway between the entorhinal cortex and PC (Whitlock et al., 2008), the present results quantitatively confirm that this route provides weak inputs to the PC, and that an alternative route likely conveys the bulk of spatial information from the PC to hippocampal targets (Burwell and Amaral, 1998). Specifically, the density of reciprocal connectivity between PC and dorsal retrosplenial cortex is greater than entorhinal connectivity with PC. It should be noted that the entorhinal cortex reportedly sends modest inputs to the retrosplenial cortex (Wyss and Van Groen, 1992; Agster and Burwell, 2009), which may indirectly influence spatial processing in PC. The strong bidirectional parietal-retrosplenial connectivity reported in the present work may also subserve allocentric/egocentric coordinate transformations as has been argued in computational and experimental work (McNaughton et al., 1994, 1995; Byrne and Becker, 2007; Burgess, 2008; Vann et al., 2009; Wilber et al., 2014). Preliminary observations by Nitz and colleagues (Alexander and Nitz, 2013) have directly linked the rat dorsal retrosplenial cortex to processing allocentric and egocentric representations, supporting the notion that this region, along with the PC and hippocampus, are part of a coordinate transformation network, possible for multiple reference frames (e.g., body centered, route-centered, world-centered).

Finally, it is important to point out that several electrophysiology studies in behaving rodents have now identified neurons in the rodent PC that fire as a function of an animal's allocentric heading in an environment, known as head direction cells (Chen et al., 1994a,b; Wilber et al., 2014). Head direction cells have been observed throughout the limbic system (reviewed in Taube, 2007; Yoder et al., 2011; Clark and Taube, 2012), and in the present study, we report that some of these limbic regions, including the retrosplenial cortex (Cho and Sharp, 2001), laterodorsal thalamus (Mizumori and Williams, 1993), and anteroventral thalamus (Tsanov et al., 2011) project heavily to the PC. Thus, it is possible that head direction information could be conveyed to the PC through one or more cortical and thalamic pathways. One interesting observation in the present study is that medial portions of the PC receive the highest density of these projections. Although directionally modulated PC cells have been reported in lateral regions of the PC (Wilber et al., 2014), it is possible that directional modulation of PC cells follows a similar medial to lateral gradient. Finally, it is important to point out an alternative possibility that head direction cellular responses in the PC could be generated intrinsically through an angular path integration process involving the high-density of angular head velocity modulated cells that have been identified in the PC, in addition to those that are modulated by both angular head velocity and head direction (McNaughton et al., 1994; Whitlock et al., 2012; Wilber et al., 2014).

To summarize, the present study was directed at mapping the inputs and outputs of the region traditionally described as rat PtA, and a poorly examined region of the PC traditionally referred to as medial secondary visual cortex (Paxinos and Watson, 2007: V2MM and V2ML; Wilber et al., 2014). We recently demonstrated that this posterior zone of PC contains cell types similar to primate posterior PC (Wilber et al., 2014), and the present study suggests that the connectivity pattern of PtA is highly similar to V2M. We also demonstrate that projections to the PC vary as a function of medial to lateral location. Specifically, LPtA/V2ML have significantly different connectivity than MPtA/V2MM. Thus, similar to primates, the parietal cortex of rats exhibits a gradual shift in connectivity along the medial-lateral axis, which may represent functionally distinct areas.

### Conflict of interest statement

The authors declare that the research was conducted in the absence of any commercial or financial relationships that could be construed as a potential conflict of interest.
