# Supplementary material for: Cortical connectivity maps reveal anatomically distinct areas in the parietal cortex of the rat
Source: Front Neural Circuits. 2015 Jan 5;8:146. doi: 10.3389/fncir.2014.00146 (PMC4283643; doi:10.3389/fncir.2014.00146)
Supplement: Supplementary file 1 [file Image1.PDF]

Supplementary Material

Cortical connectivity maps reveal anatomically distinct areas in the parietal cortex of the rat

Abbreviated Title: Whole-brain rat parietal cortex connectivity

Authors: Aaron A. Wilber[1,2,4]*, Benjamin J. Clark[1,3,4]*, Alexis J. Demecha[1], Lilia Mesina[1], Jessica M. Vos[1], and Bruce L. McNaughton[1,2]

Affiliations:
[1]Canadian Centre for Behavioural Neuroscience, The University of Lethbridge, Lethbridge, AB, Canada

[2]Department of Neurobiology and Behavior, University of California, Irvine, CA, USA

Correspondence:
awilber@uci.edu
bnjclark@unm.edu
Drs. Aaron Wilber and Benjamin Clark
Canadian Centre for Behavioural Neuroscience
University of Lethbridge
4401 University Drive W
Lethbridge, AB T1K 3M4

[3]**Current Address:**
Department of Psychology, The University of New Mexico, Albuquerque, NM, USA 87131

[4]**Contributed Equally**

1. Supplementary Figures

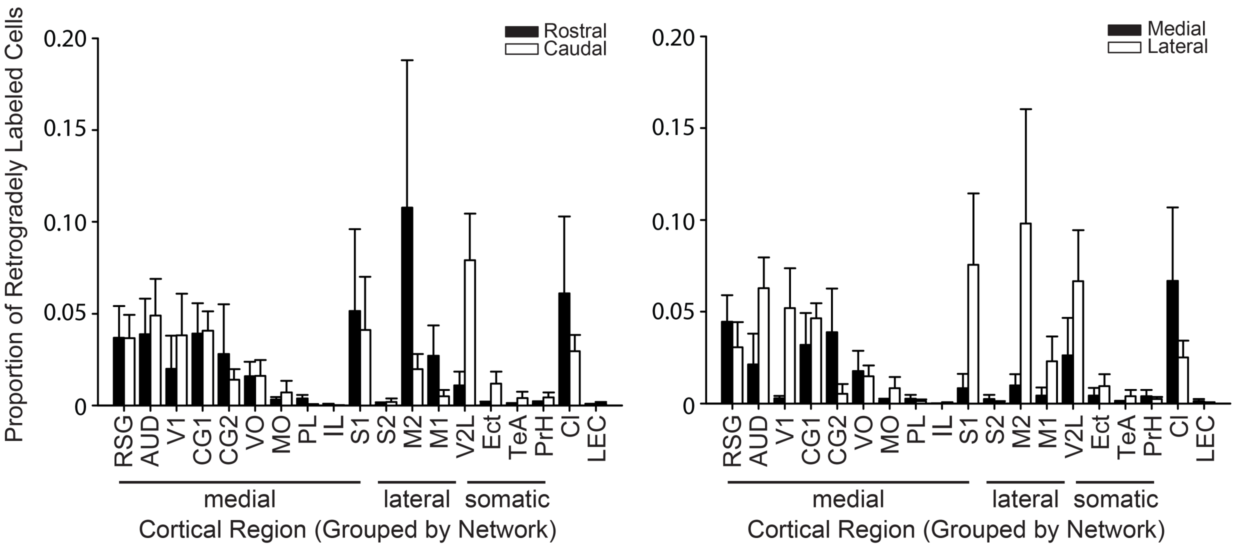

Supplementary Figure 1. Cortical inputs to parietal cortex vary as a function of injection location along the medial-lateral but not rostral-caudal axis. Bar plots showing the proportion of retrogradely labeled cells for regions in animals with injections in rostral vs. caudal parietal cortex (left panel) and medial vs. lateral parietal cortex (right panel). Data is the same as in Figure 6; however, the two regions with the highest density inputs to parietal cortex (V2M/PtA and RSD) are excluded and the remaining regions are rescaled. The split medial versus lateral and rostral vs caudal grouping was made based on anatomical boundaries. For example, PtA centered injections were classified as rostral and V2M centered injections were classified as caudal. Rescaling the lower density inputs to parietal cortex in this way further illustrates the significant medial-lateral differences and lack of rostral-caudal differences in the parietal cortex.
